# Thermal compaction as an alternative approach for full-RAP base layer construction

**DOI:** 10.1038/s41598-025-01341-3

**Published:** 2025-05-12

**Authors:** Lisley Madeira Coelho, Belayne Zanini Marchi, Pedro Henrique Poubel Mendonça da Silveira, Afonso Rangel Garcez de Azevedo, Sergio Neves Monteiro, Antônio Carlos Rodrigues Guimarães

**Affiliations:** 1 Department of Fortification and Construction, Military Institute of Engineering - IME, Praça General Tibúrcio, 80, Praia Vermelha, Urca, Rio de Janeiro, RJ CEP 22290-270 Brazil; 2 Department of Materials Science, Military Institute of Engineering - IME, Praça General Tibúrcio, 80, Praia Vermelha, Urca, Rio de Janeiro, RJ CEP 22290-270 Brazil; 3 Civil Engineering Laboratory, North Fluminense State University (UENF), Rio de Janeiro, CEP 28013-602 Brazil

**Keywords:** Reclaimed asphalt pavement (RAP), Stabilization of base, Warm base, Mechanical characterization, Permanent deformation, Civil engineering, Mechanical properties

## Abstract

The use of Reclaimed Asphalt Pavement (RAP) in road base layers represents a solution to reduce the consumption of natural aggregates. However, the variability of RAP properties poses challenges to its application, particularly regarding mechanical behavior. This study investigates thermal compaction as a strategy to stabilize mixtures composed exclusively of RAP, introducing the concept of a warm base. Repeated load triaxial tests were conducted to evaluate the effects of compaction temperature on permanent deformation (PD) and resilient modulus (RM). The results indicate that increasing the compaction temperature significantly improves the mechanical behavior of RAP, reducing PD by up to 52% at the highest stress level. Additionally, the RM of RAP-M samples increased by approximately 187.13% compared to the maximum value of RAP-F samples and 389.05% compared to the minimum value. This approach enables the application of larger quantities of RAP in pavements, ensuring good structural quality while minimizing the effects of the material’s initial variability.

## Introduction

In construction processes, it is essential to consider the integration of new techniques and alternative materials, focusing on environmental protection and sustainable development. Roadway projects, in particular, require large volumes of conventional materials for new constructions and pavement rehabilitation, contributing to the depletion of limited natural resources^1^. Aiming to meet sustainable development demands, highway engineers have been exploring the use of alternative materials, currently considered as waste^2^. The recycling and utilization of these materials can reduce the need for extraction and processing of virgin aggregates in roadway projects, providing significant environmental benefits, such as reducing energy consumption and greenhouse gas emissions during production.

Reclaimed Asphalt Pavement (RAP) is one of the residual materials that can be rationally reused in new roadway projects, provided that its behavior is properly understood. RAP is obtained through the milling or removal of asphalt layers from pavements undergoing maintenance or rehabilitation. This material consists of natural aggregates (93–97% by weight of the mixture) coated with aged bitumen, along with cured residual asphalt (3–7%)^3^.

It is reported in the literature that nearly 70% of recent studies on RAP focus on its use in asphalt layers^3–9^. However, the lack of knowledge about RAP mixture production has been recognized as one of the critical barriers to achieving higher RAP recycling rates in asphalt mixtures^10^. Undoubtedly, material properties such as RAP sources, RAP percentage, RAP binder properties, virgin binder performance grade, etc., can significantly influence the performance of RAP mixtures. This limitation in the use of RAP in asphalt layers has led to an increase in RAP stockpiles worldwide.

Given the widespread availability of this potentially sustainable material, studies have investigated the feasibility of using RAP in unbound road base or sub-base layers. The suitability of RAP for these layers has been systematically investigated by various researchers^11–18^. However, the mechanical responses and deformation characteristics of RAP in unbound pavement layers are still not fully understood due to its unique properties.

One of the main obstacles to the use of RAP, imposing limitations on its maximum content, is the lack of material homogeneity, caused by variability in the original pavement from which it came, which might combine RAP from various sources, pavement ages, states of damage, and milled layers^19^.

Due to its composition and the particular characteristics of heterogeneous gradation, the type of residual binder, the specific weight, and the source rock, RAP has a different mechanical behavior than natural aggregates. Understanding how these properties affect the elastic and plastic behavior under load is important information for the design of road pavements. RAP has a higher resilience modulus (RM) compared to virgin aggregate (VA), suggesting better performance, however, to properly understand and evaluate damage to flexible pavements, the study of permanent deformation (PD) in granular materials is one of the fundamental parameters^20–27^.

Base pavement layers, which include RAP material, are very sensitive to the accumulation of PD under repeated loads. Stolle et al.^28^ found that the addition of RAP tends to increase the accumulated PD. Thakur et al.^29^ pointed out that the PD of aggregates containing RAP increases with an increase in RAP content. Jeon et al.^30^ carried out multi-stage PD tests with 100% RAP and 100% VA, applying mean normal stresses between 147 and 200 kPa, and deviator stresses ranging from 145 to 430 kPa. They observed that RAP showed greater deformation than VA under lower stresses (e.g., =145–227 kPa q = 145–227 kPa), while under higher stresses (e.g., =380–430 kPa q = 380–430 kPa), VA exhibited greater deformation than RAP^31^Attia^30^ also performed triaxial repeated loading tests on three different specimens, namely 100% RAP, 50% RAP 50% VA, and 100% VA, and reported that a mixture of RAP and VA exhibited lower PD than a 100% VA specimen. Arshad and Ahmed^32^ came to similar conclusions in that the increase in residual cumulative strains was insignificant when the RAP contents varied from 0 to 50%. However, there are reports indicating that the PD in RAP-VA mixtures may be lower than that of pure VA, showing a minimum PD in some comparisons^31–33^. Regarding the physical properties of RAP-VA mixtures, the optimal moisture content (OMC) and maximum dry density (MDD) vary considerably, often being comparable to or even higher than those of pure VA^33–37^. The chemical and mechanical stabilization of mixtures containing RAP has been the subject of investigation, with a focus on the use of Portland cement, which contributes to the improvement of strength and RM, making the mixture suitable for applications in structural layers^38^. In addition, stabilization with emulsion, especially in cold-recycled asphalt mixtures, has shown potential in improving the RM, although it does not necessarily reduce susceptibility to PD^16,39^.

In addition to the chemical and mechanical stabilization strategies mentioned, Soleimanbeigi and Tuncer^40^ suggested thermal conditioning to enhance and improve the geotechnical properties of RAP used as a base layer or embankment fill. In other studies, thermal conditioning has been proposed as a compressibility-reducing agent in various contexts, such as in marine sediments^41^, peat^42,43^ and recycled asphalt tile mixtures^44,45^, demonstrating effectiveness in reducing the compressibility of these materials. Similarly, Yuan et al.^8^ point out that the use of high temperatures during the construction process, in the context of hot climate environments, can improve the mechanical behavior of RAP. Similarly, Coelho e Guimaraes^39^ indicate that thermal compaction, by promoting the activation of the residual binder, can make mixtures with high RAP contents more stable and even superior to VA under specific loading conditions. These findings reinforce the idea that thermal compaction can be an important differentiator in addressing the challenges associated with the variation in RAP properties, providing an innovative and sustainable approach to the stabilization of base layers for pavements.

In this context, the incorporation of RAP in unbound pavement layers has been widely studied, although the results found in the international literature still show discrepancies. While some studies indicate that the PDs of RAP-VA mixtures are significant compared to pure VA^35,46,47^, others report different results, highlighting the complexity and variability of the behavior of these mixtures^31–33^. This inconsistency highlights the need for a more in-depth characterization of RAP and its mechanical behavior under different conditions, especially temperature, as the viscosity of the asphalt binder is highly dependent on this factor^48,49^.

In the face of the growing demand for sustainable solutions in asphalt pavement construction, the careful selection of recycled materials becomes essential to ensure the proper performance of pavement layers under traffic loads. In this regard, the feasibility of using partial or full RAP has been widely assessed through laboratory studies and field experimental sections, aiming to verify its ability to meet strength and durability requirements.

In Brazil, the analysis of the structural performance of flexible pavements follows a mechanistic-empirical method, implemented in the MEDINA software, which uses dynamic triaxial tests to determine fundamental parameters such as RM and PD. These tests are essential for evaluating the mechanical response of materials under repeated loading, providing valuable support for the calibration of mathematical models that estimate their structural behavior. Understanding how these properties influence the elastic and plastic behavior of materials is crucial for efficient pavement design, ensuring long-term effectiveness^50–54^.

Considering the premise presented throughout the introduction, the objective of this paper is to explore the morphological complexity of RAP and evaluate its mechanical behavior for a possible application in flexible pavement base layers. To this end, the concept of a warm base is proposed, which involves the application of thermal compaction as a stabilizing agent, aiming to improve the mechanical properties of mixtures composed exclusively of RAP, thereby increasing their resistance to PD. The evaluation of this approach was conducted through repeated load triaxial tests at various stages, allowing the analysis of the behavior of the mixtures under cyclic loading.

## Materials and methods

**Fig. 1:**
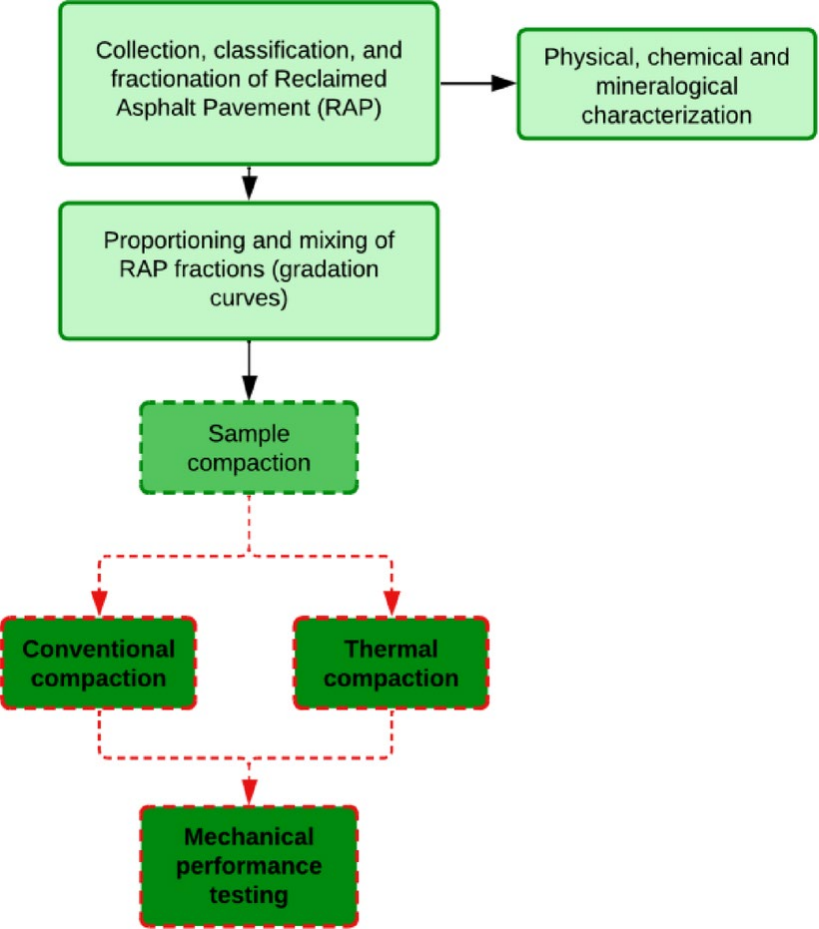
Schematic representation of the experimental procedures adopted in the study.

In order to enhance the understanding of the experimental methodology, Fig. 1 presents a schematic representation of the research workflow. The flowchart outlines the main stages of the study, including the collection and fractionation of the RAP material, mixture proportioning based on gradation, the application of conventional and thermal compaction procedures, and the subsequent mechanical performance evaluation.

### Materials

**Fig. 2:**
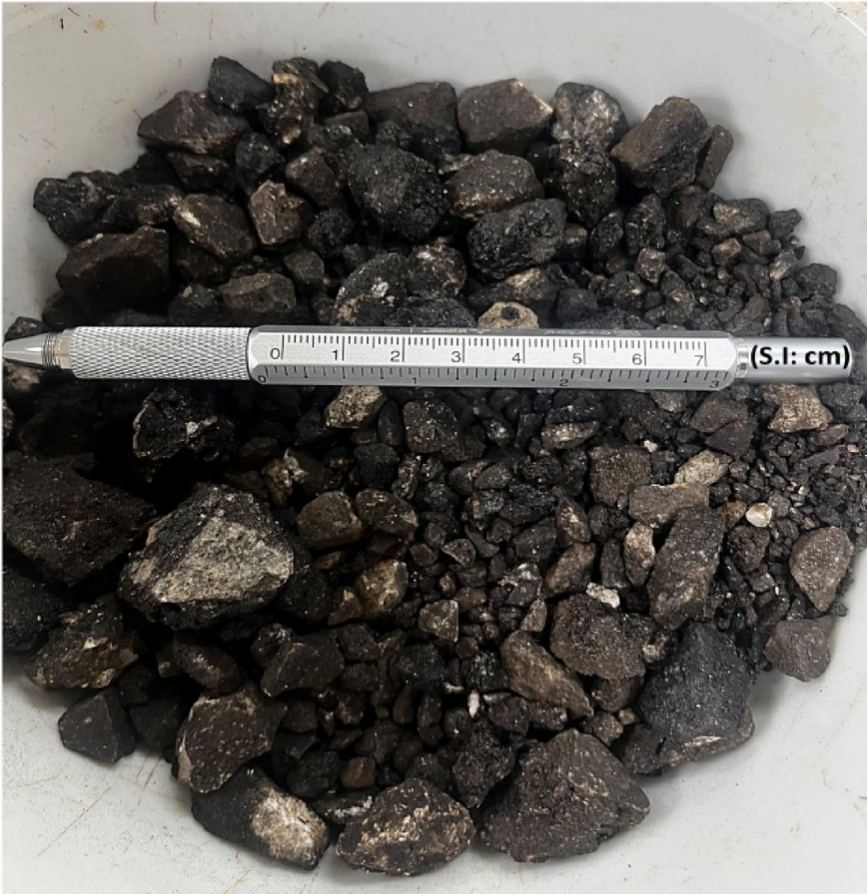
RAP samples obtained after milling from beneficiation of reclaimed asphalt.

The milled material was collected from the asphalt plant of the Municipality of Rio de Janeiro, Brazil, originating from interventions carried out in the city. The raw milled material was fractionated and divided into three samples: RAP 1 (material retained on the #3/8′′ sieve), RAP 2 (material retained on the #4 sieve), and RAP 3 (material passing through the #4 sieve). Figure 2 illustrates the visual characteristics of the processed sample.

**Fig. 3:**
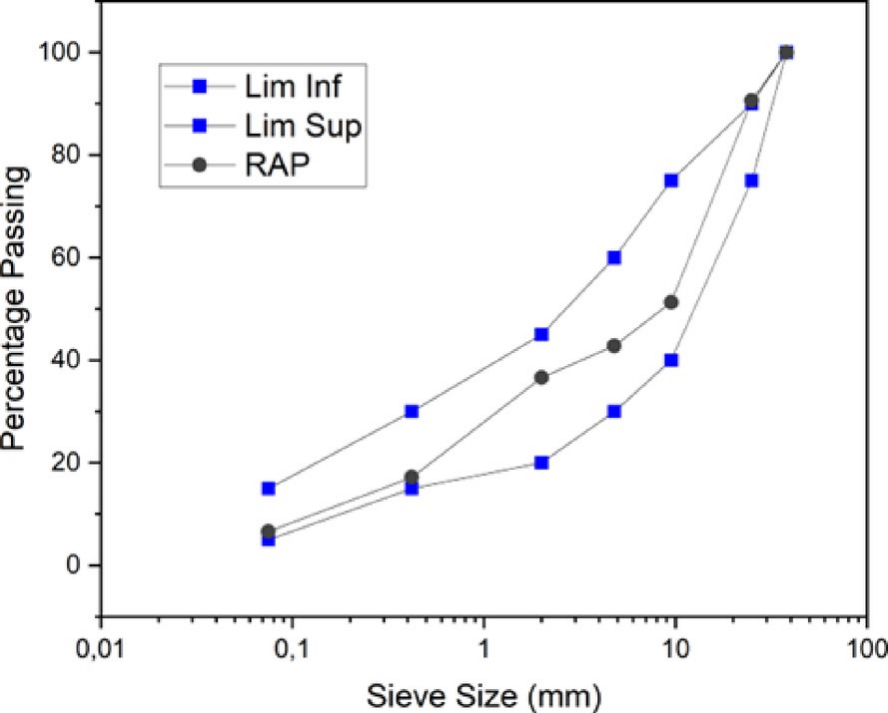
Final composition used in this study (black gradation curve).

The particle size distribution of the recycled mixture, shown in Fig. 2, with asphalt binder (black gradation curve), was obtained using the trial-and-error method, in which the goal is to fit the gradation curves in a way that allows the mixture to fall within the chosen and anticipated range specified in the Brazilian standard DNIT 141^54^ (Table 1). The mass percentage of each mixture is chosen to compose the adopted final mixture. The fitting was performed for Range B, using sieves 2′′ (50.8 mm), 1′′ (25.4 mm), 3/8′′ (9.5 mm), #4 (4.8 mm), #10 (2.0 mm), #40 (0.42 mm), and #200 (0.074 mm). Through the trial-and-error method, the particle size distribution of the recycled mixture was determined to conform to Range B of DNIT 141 standard^55^. The established composition comprises 50% RAP1, 45% RAP2, and 5% RAP3. Figure 3 shows the final composition adopted and falling within range B.

**Table 1:** Particle size distribution of RAP samples and adopted gradation curve, compared to the specification limits (Range B) from DNIT 141/^55^.

% Passing		RAP1		RAP2		RAP3				Adopted curve
								Rang B		
#(pol.)	(mm)	Sample	Attempt	Sample	Attempt	Sample	Attempt	Min	Max	
2″	50.8	100	50	100	45	100	5	100	100	100
1″	25.0	81.2	40.6	100	45	100	5	75	90	90.6
3/8″	9.5	2.5	1.25	100	45	100	5	40	75	51.25
n.º 4	4.8	1.7	0.85	82.1	36.94	99.95	4.99	30	60	42.79
n.º 10	2.0	1.6	0.8	69.4	31.23	91.3	4.56	20	45	36.59
n.º 40	0.42	1.4	0.7	33.8	15.21	25	1.25	15	30	17.16
n.º 200	0.07	0.75	0.37	13.6	6.12	1.87	0.09	5	15	6.58

### Physical, mineralogical and chemical characterization of the RAP

The properties of shape, angularity, and texture of coarse and fine RAP aggregates were analyzed using digital image processing techniques. These properties were assessed through the Aggregate Image Measurement System 2 (AIMS2), with separate analyses conducted for each nominal particle size. The system employs its methodology for classifying these materials^56^. Other physical parameter evaluated was the specific surface area by the Brunner Emmett Teller (BET). The BET was performed using a Gemini III 2375 device from Micrometrics. Dilatometric analysis were performed in a dilatometer (Netzsch, DIL, 402 E/7) with a 5 °C/min heating rate up to 150 °C, with a plateau of 1 h, in argon atmosphere (1 atm) followed by cooling with a rate of 10 °C/min.

Scanning Electron Microscopy (SEM) and Energy Dispersive X-ray Spectroscopy (EDS) were used to examine the structural arrangement of particles and qualitatively assess the chemical composition. SEM was conducted at 20 kV, with a working distance of 10.5 and 13 mm, spot size of 5, and magnifications of 400× and 1600× using a secondary electron detector. EDS compositional mapping was done with a Bruker detector integrated into the microscope. XRD analysis was performed using the Xpert Pro MRD System equipment from PANalytics with Cobalt Kα radiation (1.789 A), at a scan speed of 4°/min and a power of 40 mA × 40 kV and scanning from 20° to 55°. SEM, EDS and XRD analyses were carried out on the RAP with and without the extraction of the residual binder.

### Compactacion

Compaction is a process that promotes the rapid reduction of voids in a material by applying mechanical energy, aiming to expel the air contained between particles. This procedure is essential for establishing the relationship between the dry unit weight and the moisture content of the sample, parameters that are fundamental to the mechanical performance of materials used in pavement layers.

In the present study, the procedure described in DNIT 443^57^ was adopted, which specifies the compaction test using tripartite cylindrical molds with dimensions of 100 × 200 mm, applying the energy level equivalent to the modified proctor. This method was chosen due to its suitability for preparing specimens intended for repeated load triaxial tests, ensuring sample representativeness and uniformity. Given the granular behavior of the RAP in this study and its application in pavement base layers, the compaction procedure defined by DNIT 443^57^ was adopted, as it ensures compatibility with repeated load triaxial testing. Other methods, such as Marshall or gyratory compaction, are primarily intended for asphalt mixtures used in surface layers and are not standardized for granular materials or triaxial specimen preparation.

To assess the potential effect of heating on the mechanical performance of RAP, compaction was carried out under two different conditions: at room temperature, as commonly practiced in laboratories, and with prior heating of the mixture, aiming to activate the residual asphalt binder present in the material. This strategy represents the main innovation proposed in this study and is detailed in the following subsections.

#### Conventional compaction

Conventional compaction of the RAP samples was carried out at room temperature (25 °C), in accordance with usual laboratory practice. Following the methodology of DNIT 443^57^, the samples were molded in tripartite metal cylinders (100 mm × 200 mm), applying 21 blows per layer, totaling 10 layers each approximately 2 cm thick, in accordance with the energy corresponding to Modified Proctor.

To determine the OMC for each RAP type, a series of compaction tests was performed in which the water content was systematically varied. For each moisture condition, the MDD was calculated based on the ratio between the dry weight and the volume of the specimen. The OMC was identified as the moisture content associated with the maximum MDD. This procedure ensured consistent and representative sample preparation for the subsequent mechanical testing.

#### Thermal compaction

The thermal compaction of the RAP samples was carried out with the purpose of activating the residual asphalt binder present in the material, thereby promoting thermal stabilization during the molding process. A target temperature of 80 °C was established as a reference for this condition.

To achieve this, the loose RAP material was initially heated in an oven at 110 °C for 2 h (± 1 °C), with the temperature monitored by a thermometer inserted directly into the material. The initial overheating was adopted as a compensatory measure for the natural heat loss that occurs during layered molding, performed in 10 layers of approximately 2 cm each, with 21 blows per layer, following the modified proctor compaction energy defined by DNIT 443^57^. Compaction began once the mixture reached 90 °C, ensuring that the temperature of the specimen remained close to the 80 °C target throughout the molding process. This temperature control aimed to provide better thermal uniformity between layers and to facilitate the mobilization of the residual binder during compaction.

**Fig. 4:**
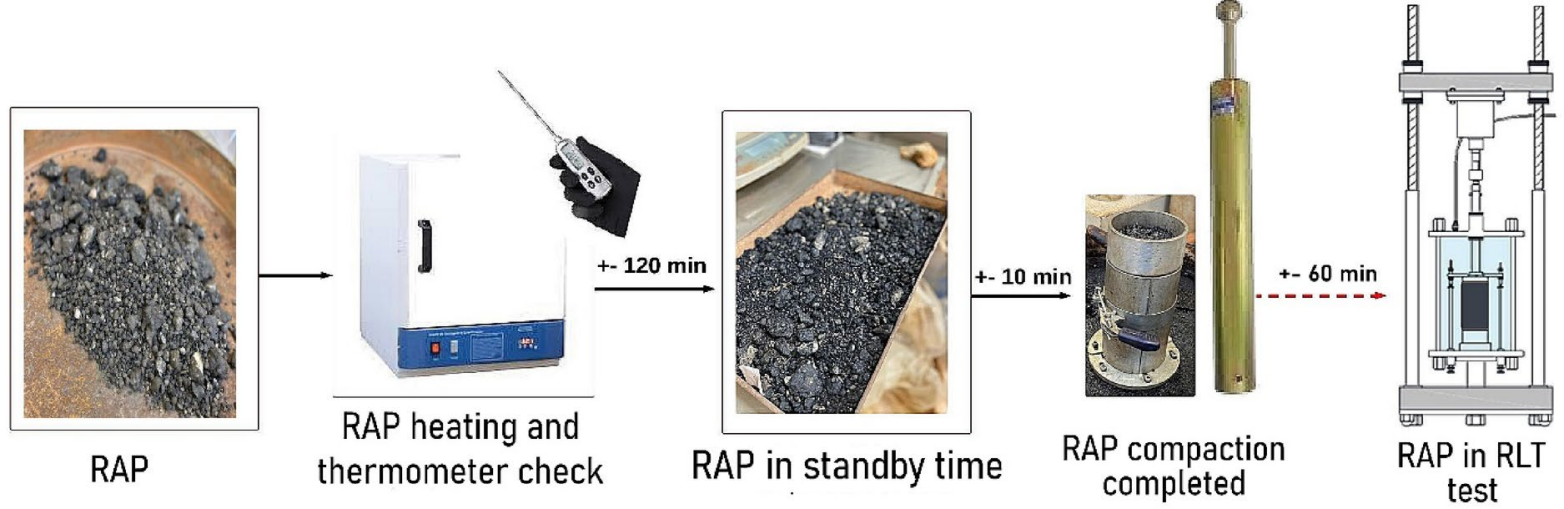
Stages of the thermal compaction process.

The compaction process lasted approximately 5 min (± 1 min), with the mixture temperature remaining within ± 1 °C of the target temperature in the intermediate layers. Figure 4 illustrates the thermal compaction process, detailing the heating, temperature control, and molding steps.

### Mechanical analysis

**Fig. 5:**
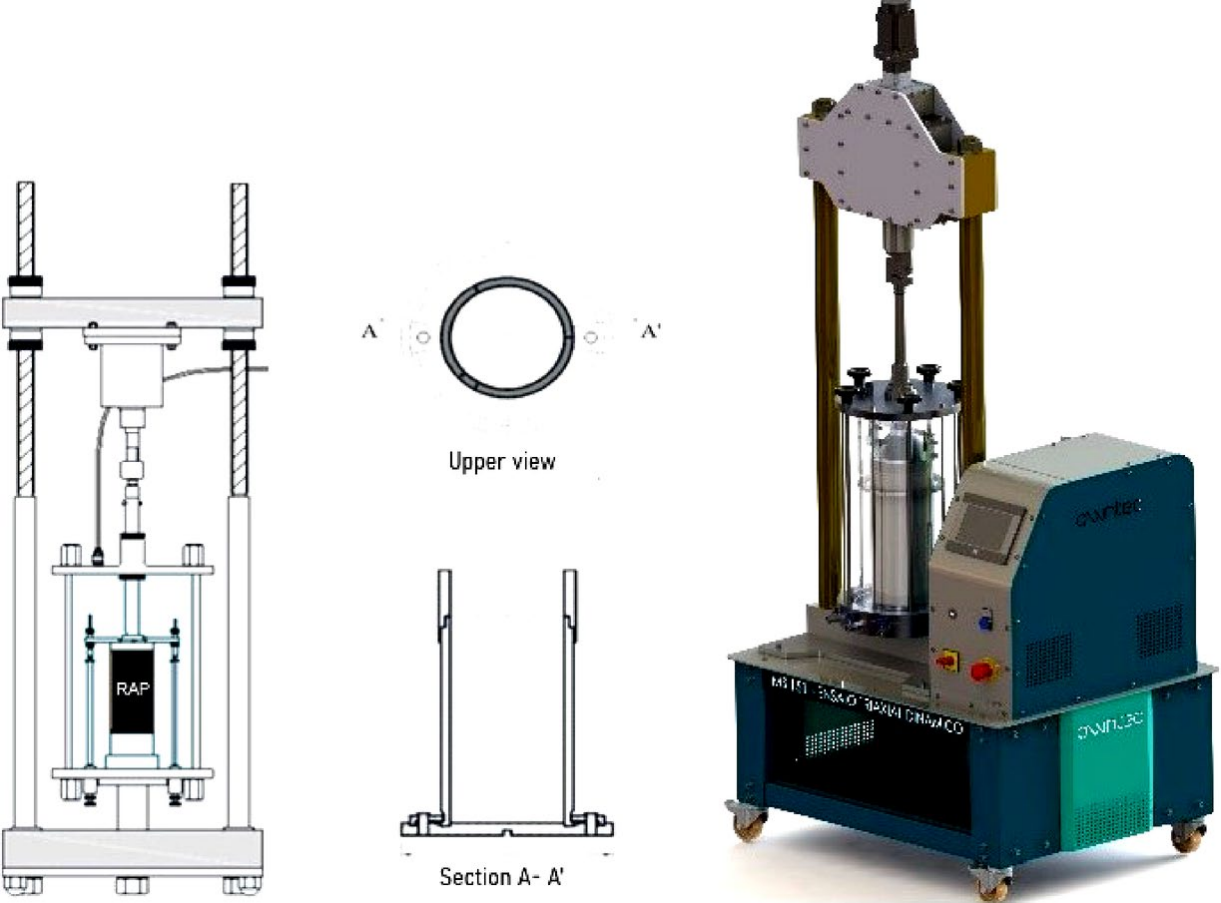
Dynamic triaxial equipment (Owntec, model MS 151) used in this study for mechanical testing of RAP specimens. Schematic adapted from technical documentation provided by Owntec.

The mechanical behavior of the RAP specimens was evaluated through repeated load triaxial (RLT) testing, conducted in accordance with standardized procedures for assessing both RM and PD in granular layers. Figure 5 illustrates the main components of the equipment used for conducting these tests, performed with a dynamic triaxial system (Owntec, model MS 151), available at the soil mechanics laboratory of the Military Institute of Engineering (IME). The setup consists of a pneumatic press, a triaxial cell or chamber, an axial load transducer, and a vertical displacement measurement system using a linear variable differential transformer (LVDT), along with a tripartite cylindrical mold with a base and two steel clamps and a complementary ring (collar), properly shown in top view and in section. All RM and PD tests on samples compacted at different temperatures were conducted at room temperature (25 °C ± 1 °C).

### RM test

The RM test depends on the nature of the soil, texture, plasticity of the fine fraction, moisture content, density, and stress state^51^. In pavement mechanics, the RM is defined as the ratio between the cyclic load applied and the elastic or recoverable deformation of the material^58^. It is a parameter aimed at characterizing the elastic behavior of materials, such as soils and aggregates, under repeated loading, either in the laboratory or by the repeated actions of vehicle loads on the pavement. The RM is obtained from the results of repeated load triaxial tests, defined as the ratio between the deviator stress (σ1 − σ3) and the axial resilient strain, Δr, which is understood as the ratio Δh by h0, where Δh is the maximum vertical displacement and h0 is the initial reference length of the cylindrical specimen. We aimed to reproduce in the laboratory the loading conditions of traffic loads on the pavement structure. Such a relationship for most pavement materials is nonlinear, unlike other elastic solids, with a strong dependence on the applied stresses^59^.

In this research, the test was conducted according to the Brazilian standard test method DNIT 134^60^, using a Brazilian-made dynamic triaxial testing machine (Owntec-Brazil, Santa Cruz do Sul, Brazil) (MS-151). During the RM test, 18 pairs of confining stresses (σ_3_) and deviator stresses (σ_d_) were applied after the specimen conditioning phase. The loading cycle duration was 1 s, with 0.1 s of loading application and a frequency of 1 Hz (60 cycles per minute). In the conditioning stage, the specimens were exposed to three sets of stresses and subjected to 500 loading cycles for each set. Subsequently, they were subjected to an additional 18 sets of stresses, with 100 loading cycles for each set, totaling 3300 cycles per test. The values of the applied stresses are presented in Table 2.

**Table 2:** Pairs of stresses for RM test.

Pair	*σ*_3_ (KPa)	*σ*_*d*_ (KPa)	*σ*_3_/*σ*_1_
Conditioning phase			
1	70	70	2
2	70	210	4
3	105	315	4
Loading phase			
1	20	20	2
2		40	3
3		60	4
4	35	35	2
5		70	3
6		105	4
7	50	50	3
8		100	2
9		150	3
10	70	70	2
11		140	3
12		210	4
13	105	105	2
14		210	3
15		315	4
16	140	140	2
17		280	3
18		420	4

### PD of the multi-stage type

Thus, in this study, the multi-stage PD test was used to assess the behavior of the RAP under a variety of stress conditions. For this particular, the European standard for triaxial testing was used as a reference^61^. The confinement stresses were estimated based on the literature. Barksdale^62^ reported that, in the compacted base pavement layer, the confinement stress is approximately 70 kPa due to the locked-in stresses resulting from compaction. The literature also states that confinement stresses in the upper half of the base layer range between 35 and 50 kPa, while, in the lower half of the base layer, stresses range from 20 to 35 kPa, but never exceed 50 kPa^63^.

Although confinement stresses above 50 kPa are not usual in the literature, the study by Allah et al.^54^ evaluated a confinement stress of 140 kPa to develop the shakedown envelope of the material. Therefore, in this research, it was deemed relevant to assess confinement stresses of 50, 70, and 100 kPa. Each stress pair (see Table 3) was applied at a frequency of 5 Hz over 5000 cycles, totaling 25,000 cycles on a single specimen, resulting in approximately 1 h and 30 min of testing time per test. The multi-stage RTL provides the foundation for understanding material behavior under a variety of loading and stress conditions with a minimal number of tests required. However, multistage RTL tests have their drawback, commonly referred to as the stress history effect, on the magnitude of total accumulated deformation^64^. Nevertheless, in this research, the objective of applying the test was to predictively and comparatively characterize the influence of compaction temperature on the mechanical behavior of recycled material in the base layer.

**Table 3:** Stress levels implemented for conducting multi-stage repeated triaxial loading.

Test nº	Stage	*σ*_*d*_	*σ*_3_	*σ*_*d*_/*σ*_3_
Test I	I	50	50	1.0
	II	75	50	1.5
	III	100	50	2.0
	IV	125	50	2,5
	V	150	50	3.0
Test II	Ia	70	70	1.0
	IIa	105	70	1.5
	IIIa	100	70	2.0
	IVa	125	70	2.5
	Va	150	70	3.0
Test III	Ib	100	100	1.0
	IIb	150	100	1.5
	IIIb	200	100	2.0
	IVb	250	100	2.5
	Vb	300	100	3.0

## Results and discussion

### EDS, SEM and XDR analysis

**Fig. 6:**
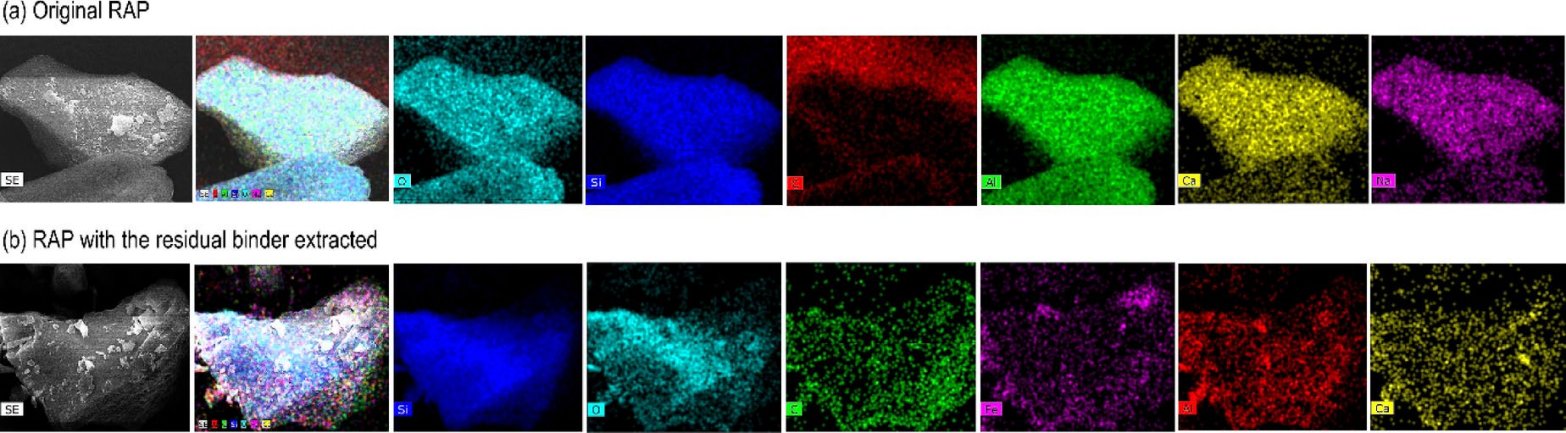
EDS results of RAP.

The original RAP (see Fig. 6a) exhibited an elemental composition of Oxygen (40.82%), Silicon (22.31%), Carbon (16.13%), Aluminum (11.31%), Calcium (5.71%), and Sodium (3.73%). In contrast, the RAP with the residual binder extracted (see Fig. 6b) showed an elemental composition of Silicon (48.24%), Oxygen (37.66%), Carbon (12.78%), Iron (0.74%), Aluminum (0.44%), and Calcium (0.13%). After the extraction of the residual binder, the carbon content in the RAP decreased from 16.13 to 12.78%, indicating the removal of the asphalt binder. Silicon, the primary component of the mineral aggregate, increased from 22.31 to 48.24%, highlighting the exposure of the mineral matrix. Reductions in oxygen (40.82–37.66%) and aluminum (11.31–0.44%) further suggest the removal of binder-associated components. The presence of iron (0.74%) in the extracted RAP, absent in the original RAP, indicates the exposure of iron minerals. The significant decrease in calcium (5.71–0.13%) and the undetectable levels of sodium (initially 3.73%) reinforce the binder’s contribution to the RAP composition.

**Fig. 7:**
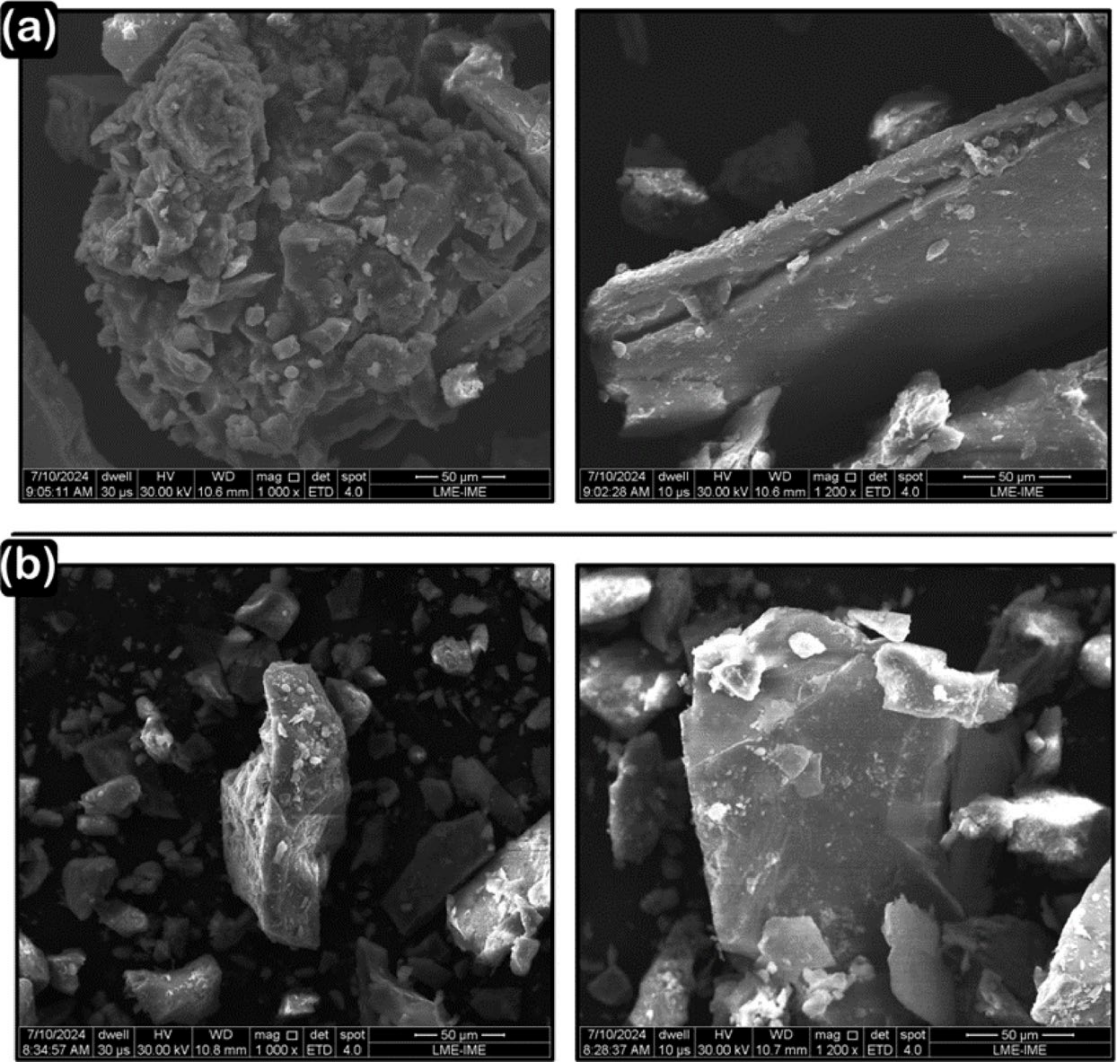
SEM of RAP particles: (**a**) Original RAP. (**b**) RAP with the extraction of the residual binder.

Figure 7 shows the SEM images of the original RAP and RAP with binder extraction, at magnifications of 1000x and 1200x, respectively. The RAP particles covered with asphalt binder exhibit a smooth, cohesive surface, with a uniform binder distribution that smooths out irregularities and acts as a hydrophobic barrier, hindering moisture absorption and achieving ideal compaction. After binder extraction, the SEM images reveal rougher and more porous surfaces, with exposed internal structure and defined morphology. The hydrophobic nature of RAP poses a challenge for water-based compaction, suggesting the need for emulsifying agents, additional VA, or adjustments in the compaction process, such as thermal stabilization.

**Fig. 8:**
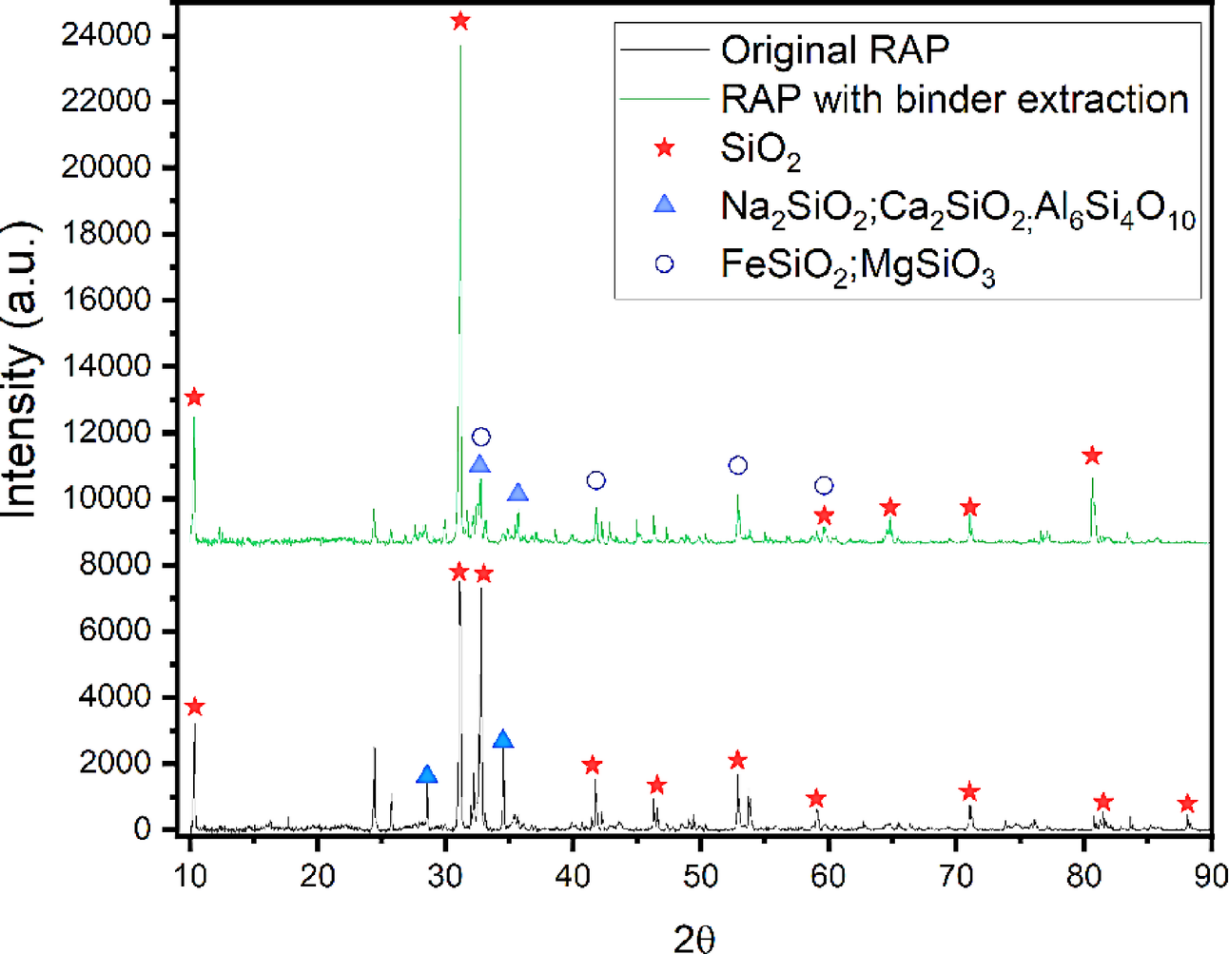
XRD results of the RAP: the lower curve represents the original RAP, while the upper curve corresponds to the RAP with binder extraction.

Figure 8 shows the XRD analysis of the original RAP, peaks of silicon dioxide (SiO2), sodium silicate (Na2SiO3), aluminum oxide (Al2O3), and calcium oxide (CaO) were identified, indicating a complex mineralogical matrix. In the RAP with binder extraction, additional peaks of iron silicate (Fe2SiO4) and magnesium silicate (MgSiO3) emerged, suggesting the formation or concentration of new mineral compounds, possibly due to recrystallization or precipitation of new materials induced by binder extraction.

The increase in silicon in the EDS analysis (from 22.31 to 48.24%) and the presence of silicon dioxide (SiO2) peaks in the XRD suggest that the original VA contains a significant amount of quartz, a common mineral in paving aggregates due to its hardness. The presence of other silicate peaks, such as sodium silicate (Na2SiO3) and iron silicate (Fe2SiO4), indicates the possible inclusion of feldspar and other types of crushed stone in the aggregate composition.

### AIMS, BET and dilatometer analysis

The physical characterization, including analyses such as angularity, shape and texture (AIMS) and specific surface area (BET), aims to understand the potential behavior and applications of the materials. These analyses help assess the aggregate distribution, quantify surface features, and determine the potential for interaction with binders or other additives, providing valuable insights into the material’s performance in various applications. The BET analysis indicated that the fine RAP sample has a specific surface area of 4770 cm²/g. This value is higher than that of bauxite residue (2617.7 cm^2^/g)^65^, but lower than that of cementitious materials such as Portland cement, whose specific surface areas range from 9000 to 10,000 cm^2^/g^66,67^. The pore size distribution revealed a predominant peak at 1.98 nm, characterizing a predominantly mesoporous structure. These results suggest that the presence of residual binder may be influencing pore accessibility, reducing the exposure of the active surface.

AIMS analysis indicated that RAP particles have moderate angularity (sub-rounded), ranging from 37.7 to 85%, angular sphericity between 50.0% and 66.7%, and shape index (2D) between 28.9% and 46.5% of moderately rounded particles. The high texture roughness (62.0–74.0%) suggests that, even with binder coverage, the original particle texture is visible. This morphology is consistent with the results from SEM, EDS, and XRD, which reveal a homogeneous matrix influenced by mineralogical components such as SiO2, Na2SiO3, Al2O3, and CaO. The chemical and mineralogical composition of RAP contributes to a resilient structure with good properties for moisture and degradation protection.

AIMS analysis morphology is consistent with the results from SEM, EDS, and XRD, which reveal a homogeneous matrix influenced by mineralogical components such as SiO2, Na2SiO3, Al2O3, and CaO. The chemical and mineralogical composition of RAP contributes to a resilient structure with good properties for moisture and degradation protection.

**Fig. 9:**
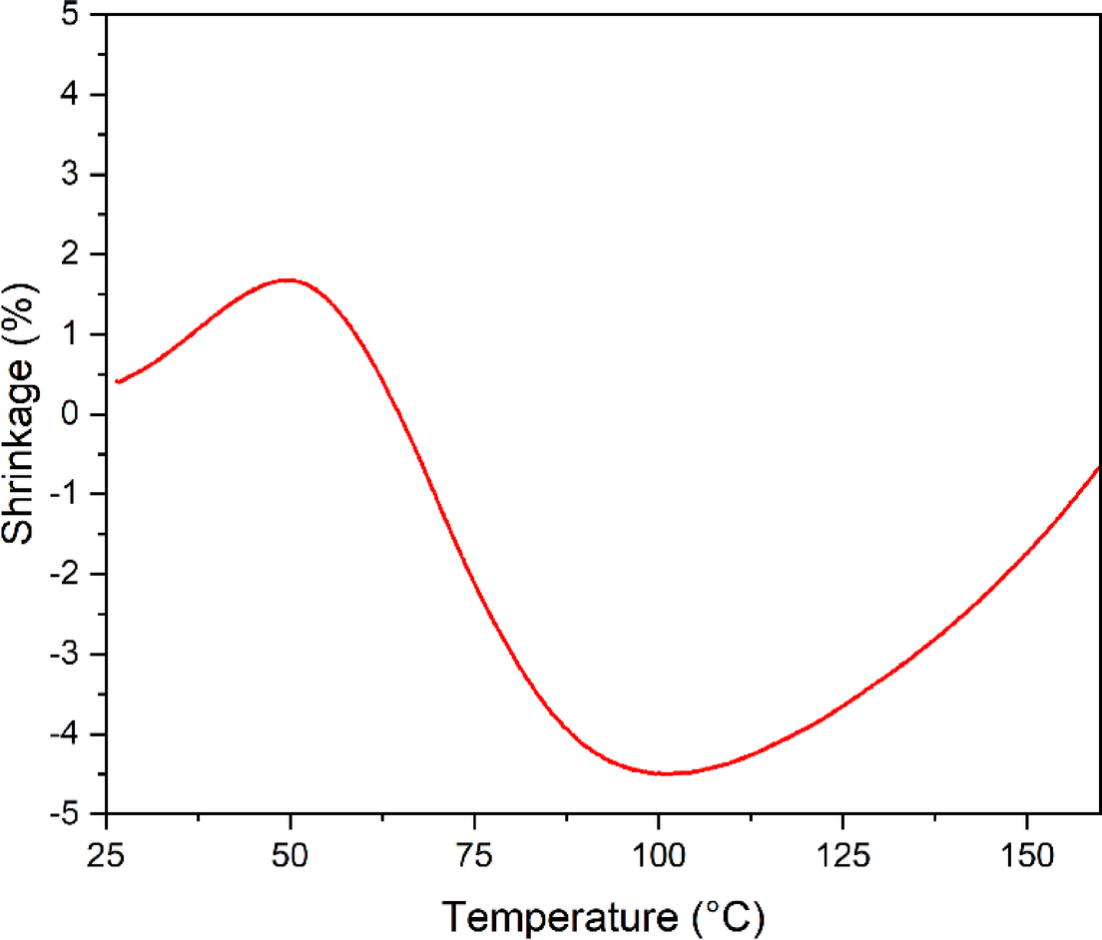
Shrinkage variation (%) as a function of temperature (°C), obtained from the dilatometry test performed on the fine fraction of RAP.

The influence of temperature on the volumetric behavior of the fine fraction of RAP was analyzed through the dilatometry test, as illustrated in Fig. 9. A slight initial expansion of the material is observed up to approximately 50 °C, followed by a sharp contraction between 50 °C and 100 °C, reaching a minimum value of around − 4% near 100 °C. Above this temperature, there is a tendency for partial volumetric recovery.

This behavior indicates that at the range of 80 °C, the temperature adopted for thermal compaction in this study, the material is in a transition stage, where fine particles are redistributed, and a possible thermal activation of the residual binder present in RAP occurs. The reduction in contraction between 50 °C and 100 °C may be associated with the loss of residual moisture and the partial softening of the asphalt binder, factors that promote compaction and consequently contribute to the increased mechanical strength of the mixture.

Thus, the results show that thermal exposure alters the internal structure of the material, promoting particle rearrangement and activation of the residual binder. In this study, thermal compaction at the target temperature of 80 °C can help accommodate the particles more efficiently.

### Mechanical analysis

#### Compaction

The RAP sample compacted with 4.54% added water presented a MDD of 1.92 g/cm^3^, while the heated sample, compacted without water addition, resulted in a slightly lower density of 1.86 g/cm^3^. Although this difference is small, it highlights the sensitive and multifactorial behavior of RAP under varying preparation conditions.

It is important to note that RAP, due to the presence of aged asphalt binder embedded in the aggregate structure, does not contain measurable free water as observed in untreated soils or granular materials. In the present study, preliminary centrifuge extraction tests were carried out using a Rotarex centrifuge, indicating residual binder contents ranging from 4.5 to 5.3% by weight. The heating process led to the softening and partial redistribution of this binder, along with possible mass loss due to evaporation of any remaining moisture. Given the heterogeneous nature of RAP, such thermal and structural transformations directly affect the internal arrangement of particles and, consequently, the resulting density after compaction.

These observations reinforce the complex and volatile behavior of the residual binder in RAP and demonstrate that small variations in laboratory procedures can lead to distinct physical responses, even within the same material batch. Furthermore, the measured densities are consistent with the range typically reported for cold recycled asphalt mixtures, which varies between 1.80 and 2.25 g/cm^3^, depending on the emulsion content, RAP percentage, natural aggregate inclusion, and compaction method^15,68,69^.

#### RM analysis

**Fig. 10:**
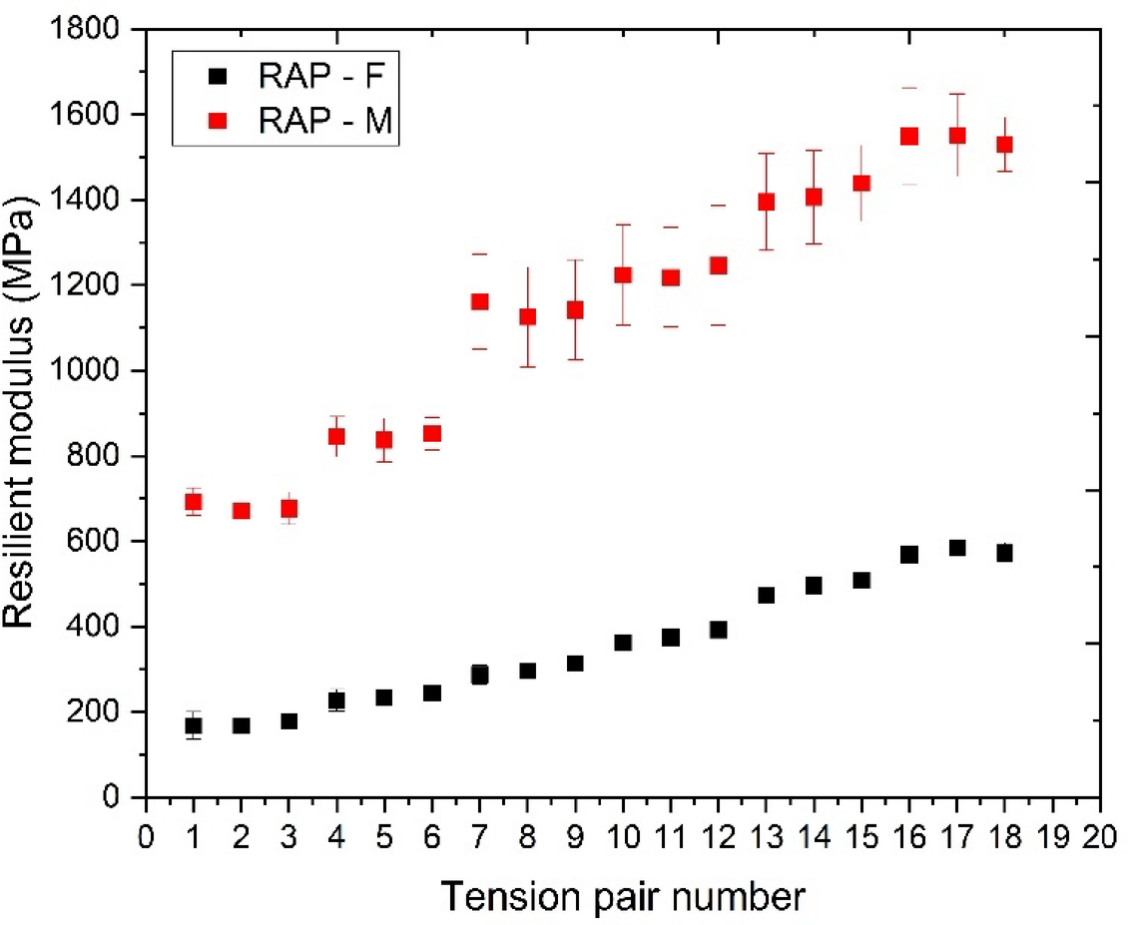
RM values of 100% RAP mixtures at different compaction temperatures.

Building upon these findings, the RM test was conducted to evaluate the stiffness of RAP samples under different compaction conditions, further assessing the mechanical effects of thermal stabilization. The analysis of Fig. 10 reveals significant differences between the RAP-M samples, which underwent thermal stabilization, and the RAP-F samples, which were not heated. The RAP-M samples exhibited a modulus range from 715.24 MPa to 1663.04 MPa, while the RAP-F samples showed a narrower range from 146.03 MPa to 581.11 MPa. These results align with the dilatometry observations, reinforcing the hypothesis that thermal compaction contributes to improved mechanical performance by optimizing particle interaction and enhancing binder softening.

The RM of the RAP-M samples increased by approximately 187.13% compared to the maximum value of the RAP-F samples and by 389.05% compared to the minimum value of the RAP-F samples. The significant increase in modulus values can be attributed to the thermal stabilization process, which resulted in higher modulus values and greater variability in the data. The observed variability in the RAP-M samples may be due to inconsistencies in the thermal stabilization conditions, such as uneven heat distribution and the material’s response to temperature changes. Nevertheless, the thermal stabilization likely induced modifications in the RAP structure, enhancing the cohesion between aggregates and the residual asphalt binder, leading to a stiffer response.

In line with these findings, the results of this study confirm the expected behavior of RAP under thermal stabilization conditions. While direct comparisons with VA were not conducted, the observed increase in RM values for RAP- M samples aligns with the anticipated performance improvements typically associated with RAP^20–23^, demonstrating its potential to enhance the stiffness and overall stability of pavement layers.

#### PD analysis

**Fig. 11:**
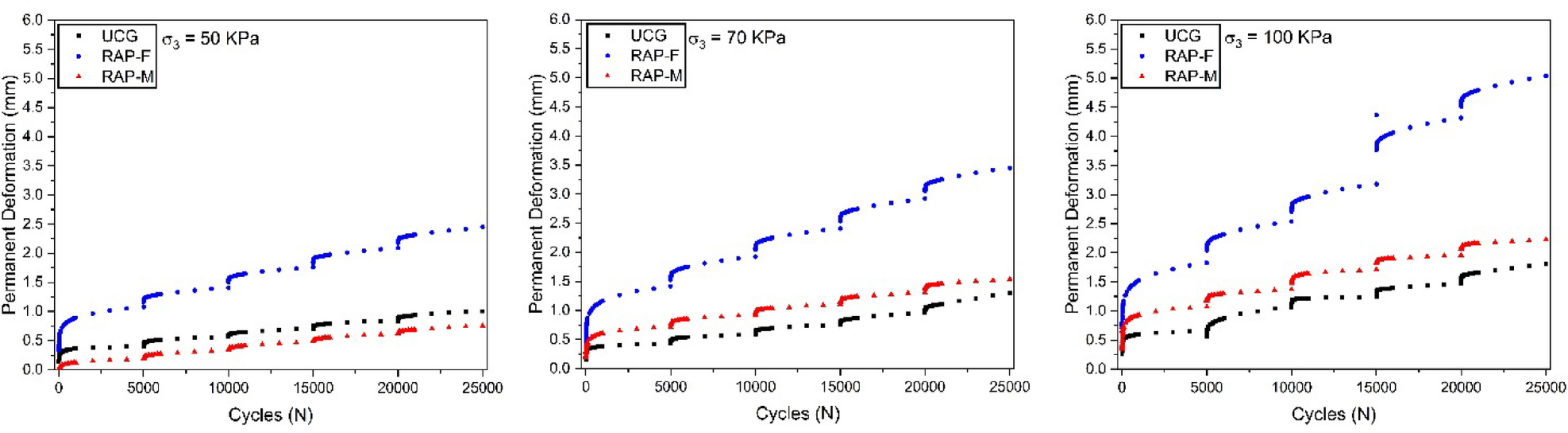
PD results for the studied materials under fixed confining stresses of 50, 70, and 100 kPa (σ_3_ = 50, 70, and 100 kPa). Comparison between ungraded crushed gravel (UCG), cold-compacted RAP (RAP-F), and heated-compacted RAP (RAP-M).

For comparative purposes in the analysis of PD, we used ungraded crushed gravel (UCG), composed of 100% VA sourced from the Mato Grosso do Sul region, Brazil. This material, employed as a base layer at the Dourados airport, serves as a reference for granular material behavior to evaluate the use of RAP, a recycled material, against a natural VA. Figure 11 presents the PD analysis results under fixed confining stresses of 50, 70, and 100 kPa, as indicated by the symbols σ_3_ = 50, 70, and 100 kPa, respectively. These values were selected based on British standards and allow for a comparative assessment between RAP-F and RAP-M while also examining their mechanical behavior relative to UCG. The RAP-M, compacted with heating, represents the warm base approach adopted in this study.

The maximum observed PD was 2.2 mm in the test conducted under a confining stress of 100 kPa. This value represents the contribution of RAP-M to the total rut depth in a pavement composed of this material. Considering an admissible deformation limit of 10 mm, the corresponding contribution percentage would be 22%.

Furthermore, the PD values of RAP-M and UCG from Dourados are quite similar. However, RAP-M exhibits a more pronounced increase in PD as the stress level rises, reaching up to 27% higher deformation at the highest stress level compared to crushed gravel. Conversely, at lower stress levels, UCG shows a 42% increase in deformation. Additionally, the total PD values observed for RAP-M under these conditions remain below 1.0 mm, indicating that its influence on potential rutting in pavement applications would be minimal.

Finally, regarding the influence of compaction temperature on the behavior of RAP samples, thermal compaction was found to reduce PD by up to 52% at the highest stress level.

## Conclusions

This study evaluated the mechanical behavior of 100% RAP mixtures under different compaction conditions, focusing on the impact of thermal compaction on PD and RM. The results demonstrated that applying heat during specimen preparation leads to significant improvements in the mechanical properties of the material.

The heated samples (RAP-M) exhibited RM values ranging from 715.24 MPa to 1663.04 MPa, whereas the cold-compacted samples (RAP-F) showed values between 146.03 MPa and 581.11 MPa. This substantial increase confirms the positive effect of heat on particle interaction and the partial activation of the residual binder. Despite the inherent variability of RAP, the results indicate a clear trend of increased stiffness associated with thermal compaction.

Regarding PD, RAP-M demonstrated comparable or superior behavior to ungraded crushed gravel (UCG) at low confining stress levels (σ_3_ = 50 kPa), with values below 1.0 mm. At higher stress levels (σ_3_ = 100 kPa), PD increased, but remained within acceptable limits. Compared to RAP-F, thermal compaction reduced PD by up to 52%, confirming its effectiveness as a physical stabilization method.

These findings support the technical feasibility of the warm base approach, which consists of reusing pure RAP in the base layers of flexible pavements by applying heat during compaction. This method eliminates the need for chemical additives or stabilizers and represents a technically sound and potentially large-scale solution. Heating can be performed in a controlled manner using simple techniques compatible with existing pavement construction practices, such as warm mix production or mobile thermal equipment. As such, thermal compaction emerges as a sustainable, efficient, and economically attractive alternative, enabling broader use of RAP in structural layers, especially unbound granular bases, while maximizing the performance contribution of the residual binder.

## Data Availability

All data generated or analyzed during this study are included in this published article.
